# Pb(II) Ion Removal Potential in Chemically Modified *Ziziphus joazeiro* Barks

**DOI:** 10.3390/ijerph192316283

**Published:** 2022-12-05

**Authors:** Yannice Santos, Gilvânia Costa, Jorge Menezes, Alex Feitosa, Henrique Coutinho, Diniz Sena, Francisco Filho, Raimundo Teixeira

**Affiliations:** 1 Environmental and Sanitary Engineering Course, Federal Institute of Education, Science and Technology—Campus Juazeiro do Norte, Juazeiro do Norte 63048-080, Brazil; 2 Department of Biological Chemistry, Regional University of Cariri, Crato 63105-010, Brazil; 3 Science and Technology Center, Federal University of Cariri, Juazeiro do Norte 63048-080, Brazil

**Keywords:** *Ziziphus joazeiro*, adsorption, heavy metal, toxic metal, lignocellulosic, isotherm, Elovich

## Abstract

In this study, five types of modified *Ziziphus joazeiro* barks were investigated for the removal of Pb(II) ions from aqueous solutions. The samples tested were natural barks, natural powder, washed with water, ethanol at 80% (EE) and 0.5 N NaOH. Batch kinetics experiments were performed under the conditions: 24–25 °C, pH 5.5–5.8, 102 mg·L^−1^ Pb(NO_3_)_2_, 100 rpm and 0.1 g of adsorbent, and analyses of pHpzc and Fourier transform infrared spectroscopy. All adsorbents tested showed potential to remove Pb(II) ions, but the adsorbent washed by 0.5 N NaOH obtained the highest experimental performance (25.5 mg·g^−1^ at 30 min), while the EE had the least performance (20.4 mg·g^−1^ at 60 min), and maximum removals of 99.9%. The kinetic models pointed to a probable chemisorption due to the best fit of pseudo-second order and Elovich, and Boyd’s model, suggesting that intraparticle diffusion limits the adsorption until the initial minutes of contact. The Langmuir isotherm fitted better to the experimental data for the NaOH adsorbent, with maximum adsorption capacity equal to 62.5 mg·g^−1^, although the Temkin model partially fitted, both suggesting the occurrence of chemisorption. The adsorption process is reversible (>81% at 20 min) and hence the adsorbents can be recycled and the Pb(II) ions recovered.

## 1. Introduction

Belonging to the Rhamnaceae Family, *Ziziphus joazeiro* is a tree naturally present in Brazil, known mainly in the northeastern semiarid region—in Caatinga biome as “Enjoá”, “joá”, “joazeiro” [1]. The joazeiro’s fruit is used for animal and human food, and its other parts for energy purposes (firewood production), traditional medicine by the local population where the leaves, inner bark and roots are used to treat fever, bacterial infections, gingivitis, respiratory diseases and for other purposes [2], and cosmetic purposes because the trunk cortex and leaves are rich in saponin, which has great detergent value, being widely used in anti-dandruff shampoo, hair tonic and in the composition of soaps and toothpastes [3,4,5]. Nowadays, several local communities still use this plant for personal hygiene [6]; it is easy to find the crushed bark powder and the dried leaves at popular natural product markets.

The triterpene saponin substance represents a large percentage of the plant composition, reaching percentages ranging from 2 to 10% (*w*/*w*) in the bark (jujubosides), and chemically it is referred to as triterpene or steroidal glycoside [7,8]. 

Saponin is in high demand by industry and has several properties, such as physicochemical (foam production, emulsification, solubilization, sweetness and bitterness) and biological (hemolytic, antimicrobial, insecticide and molluscicide) properties that are commercially exploited in applications such as food, cosmetics, pharmaceuticals and bioremediation [9]. Due to deficiencies in the extraction process, it is very common for residues to still have certain concentrations of saponins in their composition.

Generally, the adsorptive studies using vegetal biomass as an adsorbent occur in research that produces activated carbons in physically or chemically modified versions. On the other hand, recent studies with efficient results have been carried out applying chemical modifications in the presence of acids or alkalis, compounds of magnetic nature and the participation of artificial surfactants. 

The application of surfactants improves the surface properties of adsorbents for the removal of toxic metals and increases surface charge density, electrostatic interactions and ion exchange, and also increases the rate of adsorption by creating new functional groups, in addition to increasing the surface area and volume of pores, improving the surface properties of the modified adsorbents when compared to conventional ones [10].

Among many vegetal options, the genus Ziziphus is still discreetly explored, due to its medicinal and cosmetic benefits, but it still shows promising results. A brief literary survey points out six leading species of adsorptive studies, namely: *Ziziphus spina-christi* L. [11,12,13,14,15]; *Ziziphus mauritiana*, *Ziziphus jujube* or *Zizipus jujuba* [16,17,18,19,20,21,22]; *Ziziphus lotus* [23,24,25,26] and *Ziziphus vulgaris* [27].

Others studies just cite the popular name of the seed known as “jujube or jujuba fruit” or “jujube seed” [28,29,30,31,32,33,34,35,36], a food commonly present and consumed in the Mediterranean region [18]. From this compilation, the seeds and their derivatives (husks and pits) are the most investigated parts, and then the leaves, branches and trunk bark.

The lignocellulosic raw materials have good specific surface area and the presence of functional organic groups and mineral content that allow interaction with various pollutants, from toxic metals to dyes and organic compounds [23,35,37,38,39,40,41,42]. The bark of *Ziziphus joazeiro*, in addition to presenting the adsorptive advantages of plant material, also has the presence of the functional groups of the structures of the saponins, which may be active sites for interaction with pollutants.

The Pb(II) ion is considered as one of the most dangerous toxic metals for the ecosystem and is frequently studied in adsorptive assays testing raw or chemically modified vegetal materials [43,44,45,46,47,48,49]. Its removal from effluents produced from activities in the manufacture of ceramics, paints, plastics, automotive parts and batteries is extremely important to avoid problems in the aquatic environment, due to its bio-accumulative, non-biodegradable characteristics and its toxicity to plants and animals occurring even at low concentrations [50,51,52]. The effects and consequences of exposure to low and high doses of lead include headaches, stomach and muscle pain, fatigue and vomiting, anemia, liver damage, nervous system disorders, cognitive problems such as memory loss and psychological and reproductive issues [53,54].

In this study, we report the adsorptive potential of *Ziziphus joazeiro* barks before and after chemical changes, for the removal of the Pb(II) ion, through the analysis of kinetic and equilibrium adsorptions. 

## 2. Materials and Methods

### 2.1. Collection and Preparation of Adsorbents

The raw material of the research was obtained in the Public Market of Juazeiro do Norte-Ceará-Brazil, in its commercial form, popularly known as “rasp of Juá”. In Environmental and Sanitary Engineering Laboratory, Federal Institute of Ceará, campus Juazeiro do Norte, the entire sample was manually macerated in portions of particle size between 1.180 and 300 µm. From these portions, the influence of the different treatments was tested, through washes: with water, 0.5 N NaOH (alkaline treatment) and with 80% ethanol, aiming to represent domestic use, break the lignocellulosic structure, and achieve saponin extraction, respectively, promoting physical and chemical changes. A sample was also tested in its natural condition in a smaller particle size (<300 µm). In this way, the total samples obtained were five: natural, washed with water, washed with 80% ethanol, washed with 0.5 N NaOH and the last, natural powder. All the reagents used were analytical reagent grade. The treatment steps are detailed in Table 1. 

### 2.2. pH at Zero Charge (pHpzc) and FT-IR

The determination of the pH at zero charge was adapted from the methodology [55], in which 0.1 g of each adsorbent was submitted in contact with 20 mL of solution with different pHs, ranging from 1 to 13, for 2 h, under stirring at 100 rpm on a Ethik technology model 109-1 shaker (São Paulo, Brazil). The O pHpzc is calculated by plotting a graph of initial pH versus final pH, and then determining the average pH located in the buffer region.

Infrared spectra were recorded using FTIR spectrometer (FT-IR Cary 660 Agilent, ATR (Ge), 16 accumulations, resolution of 4 cm^−1^, Santa Clara, CA, USA.).

### 2.3. Batch Experiment Adsorption Kinetics and Models

The kinetic tests in batch were performed using 25 mL of Pb (II) nitrate solution buffered with acetic solution at 102 mg·L^−1^ concentration and final pH 5.5–5.8, with 0.1 g adsorbent. The batch experiments were carried out in a 125 mL Erlenmeyer flask, stirring 100 rpm on a Nova Ética model 109-1 shaker, and with liquid temperature between 24–25 °C. The contact times tested were 1, 4, 8, 16, 30, 60, 120 and 240 min. The adsorptive capacity at any of these times (*qt*) of *Ziziphus joazeiro* and the removal of Pb(II) ions in percentage form were determined using Equations (1) and (2), where *C*_0_, *C_f_*, *W* and *V* are initial concentration, final concentration (mg·L^−1^), weight (g) and volume (L).

(1) $$qt\;\left(mg\cdot g^{-1}\right)=\frac{\left(C_{0}-C_{f}\right)V}{W}$$

(2) $$E\left(\%\right)=\frac{\left(C_{0}-C_{f}\right)}{C_{0}}\times 100$$

In order to investigate the rate and mechanism of the metal uptake, Pseudo-first order (PFO) Equation (3), Pseudo-second order (PSO) Equation (4) and Elovich kinetic Equation (5) nonlinear models were used to fit the data.

(3) $$qt=qe\times \left(1-e^{-k_{1}\times t}\right)$$

(4) $$qt=qe^{2}\times k_{2}\times t/1+qe\times k_{2}\times t$$

(5) $$qt=\frac{1}{\beta }\left(1+\alpha \beta t\right)$$

where *qt* and *qe* are adsorption capacity in any time and at equilibrium (mg·g^−1^); *k*_1_ is Pseudo-first order adsorption rate constant (min^−1^); *k*_2_ is Pseudo-second order adsorption rate constant (g·mg^−1^·min^−1^); α is initial adsorption rate (mg·g^−1^·min^−1^) and β is desorption constant (mg·g^−1^). 

To verify the intraparticle diffusion mechanism, the Boyd model Equation was applied (6), where *F* is *qt*/*qe*, *Bt* is mathematic function *F*; *D* is coefficient for effective diffusion (cm^2^·min^−1^); *r* is radius particle (cm) and B is Boyd model constant (slope) equal π^2^*D*/r^2^ [30,56,57,58].

(6) $$F<0.85:\;\;\;\;\;\;Bt={\left(\sqrt{\pi }-\sqrt{(\pi -(\frac{\pi^{2}F}{3}})\right)}^{2}\;\;\mathrm{or}\;F>0.85:\;\;\;\;Bt=-0.49770-\ln\left(1-F\right)$$

### 2.4. Batch Experiments of Equilibrium Isotherms and Models

The isotherms were performed in duplicate, using 10 mL of lead (II) nitrate buffered with acetic buffer solution (final pH 5.5–5.8), and concentrations ranging from 15 to 747 mg·L^−1^, in contact with 0.05 g of adsorbent, for 30 min (equilibrium time verified in the kinetic tests), under stirring 100 rpm, and liquid temperature between 24 and 25 °C. The nonlinear models of Langmuir, Freundlich and Temkin were calculated according to the Equations (7)–(9) [59,60,61,62].

(7) $$qe=\frac{qmax\times K_{L}\times Cf}{1+K_{L}\times Cf}\;$$

(8) $$qe=K_{F}\times Cf^{\frac{1}{n}}$$

(9) $$qe=\frac{RT}{b}\ln\left(k_{T}\times c_{f}\right)$$

where *qe* is adsorption capacity at equilibrium (mg·g^−1^), *qmax* is maximum theoretical biosorption capacity for monolayer (mg·g^−1^), *K_L_* is Langmuir constant, *Cf* is ion concentration in equilibrium solution (mg·L^−1^), 1/n is adsorption intensity constant Freundlich, *K_F_* is Constant of Freundlich (mg·g^−1^)(L·mg^−1^)^1/n^, *K_T_* is constant Temkin (L·mg^−1^), *R*: Universal gas constant (J·mol^−1^·K^−1^), *T*: Temperature (K) and *b* is heat of adsorption (J·mol^−1^). Equations and parameters of equilibrium isotherm models were applied to experimental data only for the adsorbent washed with NaOH due to its better performance. 

The solutions collected after the kinetic and isothermal tests were filtered through fast speed filter paper and had the concentrations of the respective ion metals determined by Flame Atomic Absorption Spectroscopy (FAAS), at the UFCA Central Analytical Lab, using a Varian SpectrAA 50B spectrometer (Palo Alto, CA, USA).

### 2.5. Desorption Experiments

The regeneration experiments were carried out only for the adsorbent washed with NaOH sample from isotherm, after contact with solution 600 mg·L^−1^. The material was dried in an oven at 50 °C for 1.5 h, and desorption test conditions were: 0.05 g of adsorbent was mixed with 100 mL of 0.1 M HCl solution (duplicate), liquid temperature between 24 and 25 °C, stirring 100 rpm on a Nova Ética model 109-1 shaker and contact times of 10, 20 and 30 min. 

The solutions collected after desorption tests were filtered through fast speed filter paper and had the concentrations of the Pb(II) ions determined by Flame Atomic Absorption Spectroscopy (FAAS), as previously mentioned.

The desorption efficiency (*DE*) was calculated according to the following Equation (10), where *DE* (%) is the desorption efficiency, *C_t_* (mg·L^−1^) is the concentration of lead ions in the desorption solution at time *t* (min), *V* is volume of the desorption solution, and *m*_0_ (g) is the amount of Pb(II) adsorbed [39].

(10) $$DE=\frac{C_{t}v}{m_{0}}\times 100\%$$

### 2.6. Analysis Error Functions

In order to determine which model fits the experimental data better, the statistical functions of error analysis notably helped, since they consider the differences in residues and errors between the values of the adsorptive capacity obtained in the experiment (*qexp*) and those predicted by the models (q_model_). Therefore, these functions were calculated: adjusted R^2^ (R^2^_adj_), chi-square (χ^2^), sum of square error (SSE), mean sum residual (MSR), root mean square error (RMSE) and relative error (%) (Appendix A). The qualification of a good fit for the kinetic and isothermal models was considered the presentation of the highest values of R^2^_adj_ and the lowest values of χ^2^, SEE, RMSE and RE%.

## 3. Results and Discussion

### 3.1. pH PZC

It is essential to determine the pHpzc of adsorbents, since this parameter could favor or harm the adsorption process, depending on the pH of the solution exposed to contact with the adsorbent. The pHpzc indicates the pH where the surface of the adsorbent material remains electrically neutral or zero; thus, adsorbents in contact with pH solutions above pHpzc show the surface negatively charged, and below it, positively [63]. For the Pb(II) ion, as it is a cation, it is essential that the surface of the adsorbent is covered with negatively charged species, in other words, with the low availability of hydrogen protons [64] favoring the electrostatic attraction and removal of this metal.

The pHpzc values of the NP, WW and NaOH samples were 4.2, 4.8 and 4.1, respectively, revealing that when exposed to the acid solutions of Pb(II), their surfaces are negative, and favor the attraction of Pb(II) cations. The surface of Natural is close to neutrality due to a pHpzc equal to 5.3, and EE is partially positive (pHpzc = 5.6). Of all the adsorbents tested, EE presented the least interaction with the cations of Pb(II).

It is important to keep the pH of the synthetic Pb(II) solution below 5.5, to avoid its precipitation and implications for the results of adsorptive capacity.

### 3.2. Kinetic Adsorption

**Figure 1:**
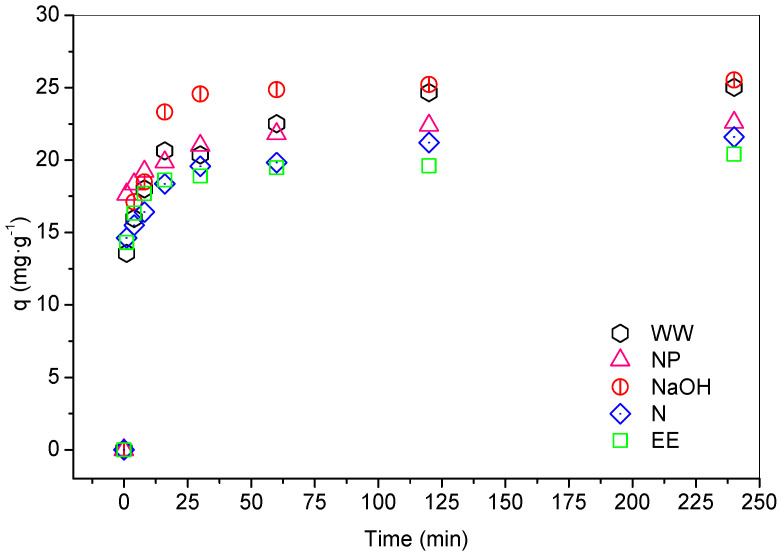
Adsorption kinetics of the five types of adsorbents tested for the removal of Pb(II) ions. Test conditions: 24–25 °C, pH 5.5 to 5.8, 102 mg·L^−1^, 100 rpm and 0.1 g of adsorbent.

Figure 1 presents the adsorption capacity (*q*) over time for the five adsorbents tested. At 30 min of contact, the NaOH and NP adsorbents achieve equilibrium, and the EE, N and the WW at the times 60 and 120 min, respectively, with variations of *qexp* less than 5% between the final times. The NaOH adsorbents reach the highest *qexp* (25.5 mg·g^−1^) and WW (25.0 mg·g^−1^), and then in sequence NP (22.6 mg·g^−1^), N (21.6 mg·g^−1^) and EE (20.4 mg·g^−1^). Regarding the removal of Pb(II) after exposure, the efficiencies found were encouraging, given that all tested adsorbents exceeded 85% removal at the best contact time, and reached 99.9% and 99.6% in the performance of NaOH and EE, respectively. 

The physical and chemical treatments had a low impact on the lead adsorption performance, due to the difference of 15.3% of the Natural adsorbent when compared to that modified with NaOH.

The use of water to wash the bark of *Z. joazeiro* is a good treatment; it presented results similar to the adsorbent washed with NaOH, but with kinetics four times slower (equilibrium time equal to 120 min). The Anova one-way test—5% significance level showed no statistical differences between the adsorption capacities of the adsorbents tested (*p*-value = 0.82, *F* = 0.368). It is also noteworthy that, although reaching equilibrium faster, the particle size of the material did not promote great improvements to the adsorptive process when compared to the natural form, just presenting the difference of 1 mg·g^−1^ in *qexp*, and 2.8% in the removal percentage. Analytically, this difference is not expressive, but in full-scale treatment processes, this percentage improvement is feasible. 

The PSO model was more adjusted than the first order one, taking as a premise the higher R^2^_adj_ values, and smaller SSE, χ^2^, RMSE and Relative Error values, which resulted in approximate data between the adsorption capacities obtained in the experiments and those predicted by the model (q_model_), and the assumption that the chemisorption process limits and controls adsorption through the valent forces of the exchange of electrons in the adsorbate adsorbent [65,66,67]. Others studies also identified chemisorption in their PSO kinetics for Pb(II) removal by testing biomass from the genus Ziziphus [16,29]. Among the adsorbents, NaOH and EE presented with the best fit (R^2^_adj_ = 0.991 and 0.986), being better than the Natural (0.942) and WW (0.933) adsorbents. 

The Elovich model describes the kinetic mechanism of the chemisorption of a solid material and adsorption on heterogeneous surfaces, and active sites with different activation energy properties, as their occupation increases [67,68,69,70,71]. Agreeing with the PSO, the Elovich model stood out for its excellent fit to the experimental data (Table 2). All R^2^_adj_ were greater than 0.994 (except NaOH—0.971), and all error functions reached their lowest level to date, certified by extremely low χ^2^ values (0.24–0.36), as well as ER (1.01–2.23) and RMSE (0.43–0.46). 

Regarding initial adsorption rate (α), the order observed was NP > EE> N > NaOH > WW, indicating that NP, EE and Natural present the highest affinity for Pb(II), although they have the lowest *qexp*. Such behavior indicates that, with the increase in the α value, lower energy is required in the adsorptive process and it occurs faster. 

Lower desorption rates (β) represent the difficulty of adsorbate desorption. The ranking WW < NaOH < N < EE < NP indicates WW and NaOH adsorbents have more difficulty in the desorption of Pb(II), and despite apparent low affinity (low β), they keep the metal ion retained, justifying the higher *qexp*. Therefore, considering the excellent fit and rankings discussed above, it is suggested that due to the affinity for the metal verified by the α, while the desorption resistance has low β, chemisorption prevails as an adsorptive process, with a probable heterogeneous surface for all, except NaOH.

Boyd’s model assumes the adsorption process occurs through the stagnant film around the external surface of the adsorbent (external or film diffusion) or intrapore (intraparticle diffusion) [30]. Once the *Bt* × *Time* graph generates a straight fit linear regression, and it still crosses the origin, it is assumed by the evaluated time range that the main resistance to mass transfer is in the diffusion inside the pores (intraparticle) [72].

**Figure 2:**
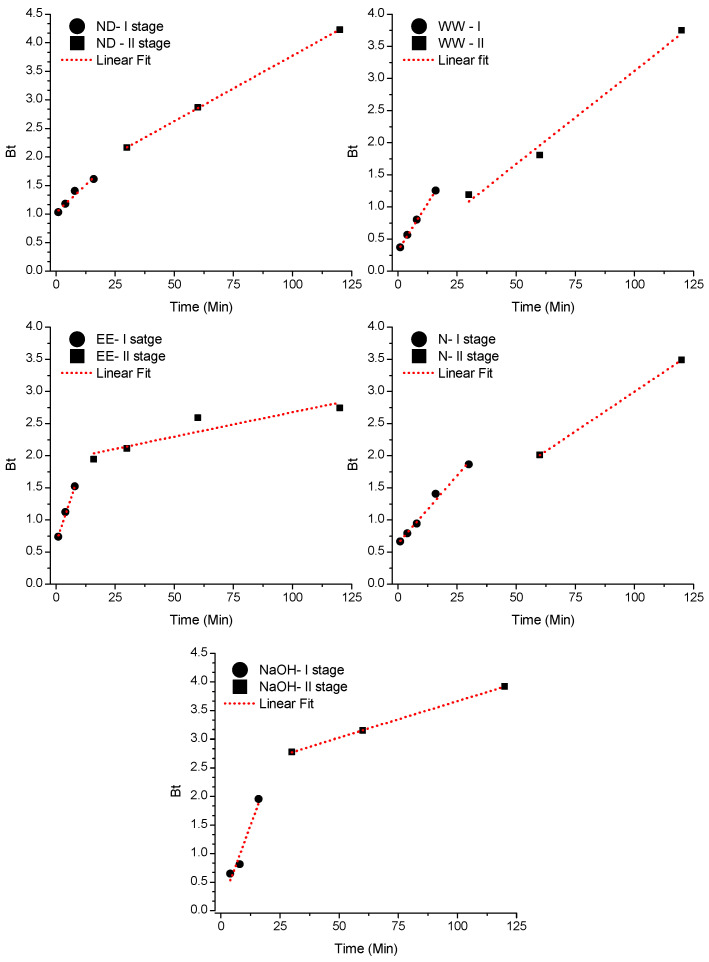
Plot *Bt* × *Time* by the Boyd linear regression diffusion model for five adsorbents tested to remove Pb(II) ions.

Table 3 shows the parameters derived from the linear fit of the model for the initial contact times (Stage I), where B is the Boyd model constant (determined by the slope of the straight), D is the coefficient for Boyd’s Effective Diffusion, and b is the linear coefficient of the straight (intercept). According to Table 3 and Figure 2, the WW, N and EE adsorbents have excellent fit and very low error, in addition to the direction of the straight pointing to origin and intercepts tending to zero. Considering the interval until the time of 16 min—WW, 8 min—EE and 30 min—N, the diffusion inside the pores has its contribution in the control of the adsorption process, with particle transit occurring without the interference of the film that was still formed [73].

After these contact times, the straight moved away from the origin, indicating the transfer resistance of Pb(II) due to the reduction of its concentration in film and occupation of the active sites on the pore surface. For the other adsorbents, it is believed that both intraparticle diffusion and external diffusion (in the film) are responsible for adsorption.

### 3.3. Adsorption Isotherms

**Figure 3:**
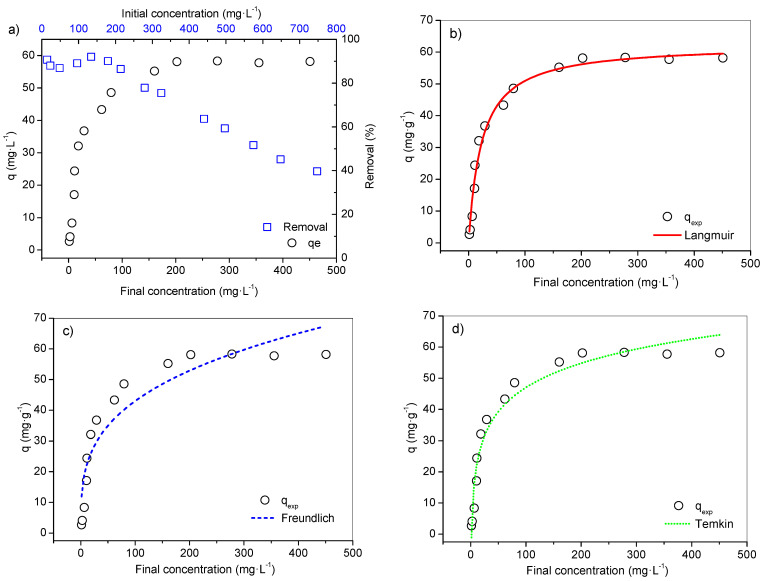
Equilibrium isotherm NaOH adsorbent under conditions 24–25 °C, pH 5.5, 30 min, 100 rpm and 0.05 g adsorbent: (**a**) Isotherm and removal percentage of Pb(II) ions from initial and final concentration. (**b**) Langmuir isotherm model. (**c**) Freundlich isotherm model. (**d**) Temkin isotherm model.

The equilibrium isotherm assay was performed testing only the adsorbent modified with 0.5 N NaOH, as it had the highest *qexp* at 30 min. The results of the isotherm analysis are presented in Figure 3. 

From this data, it possible to observe that the shape of the curve fits classification [74], such as isotherm Class L (Langmuir), subgroup II, in a scenario of surface saturation where the pollutant (adsorbent) starts to have more affinity with the solvent. Final concentrations (Cf) between 1.4 to 18.5 mg·L^−1^ involved an average removal of 89.4%. From Cf 29.0 mg·L^−1^ (*qexp* = 36.8 mg·g^−1^), the removal efficiency regressed until it reached equilibrium, and maximum experimental capacity equal to 58.3 mg·g^−1^, with initial concentrations equal to 575 and 277.9 mg·L^−1^, respectively. The removal at equilibrium reached 51.6% and, after this level, it reduced to 39.7% (Figure 3a).

According to R^2^_adj_ (0.983) and the error values from the three equilibrium models (Figure 3b–d and Table 4), the Langmuir model better describes the adsorptive separation of the metal ions by adsorbent. The *qmax* calculated by this model (62.5 mg·g^−1^) was close to the value verified in the experiment (58.3 mg·g^−1^), resulting in a relative lower error, though the isotherm data do not totally apply to the Freundlich model, and therefore it is unsafe to assume the conditions proposed by it. 

The heat of the adsorption (b) from the Temkin model was calculated as 220.80 J·mol^−1^, presenting an adjustment to a certain point to the experimental data (R^2^_adj_ 0.959), Table 4. The Temkin isotherm is based on the assumption that the heat of the adsorption is linearly decreased, and the adsorption is described by the uniform distribution of the binding energies over a number of sorption spaces [61,75,76]. This model was originally designed for a gas–solid system, but has been commonly used in adsorptive studies involving metals and is considered applicable for chemical adsorption on solid adsorbents and liquid adsorbates [62].

Therefore, the classification and good adjustments of this models point to probable chemical adsorption. As the Langmuir equation obtained the smallest error functions and better fit, this scenery suggests that the surfaces of the samples are energetically homogeneous with monolayer adsorption on a surface containing a finite number of identical sites [41,77].

The adsorption capacities of Pb(II) ions in the different types of adsorbents, modified and not, are shown in Table 5. The ability of the bark of *Z. joazeiro* modified by NaOH to remove Pb(II) ions is highlighted when comparing it to other vegetal materials from the genus Ziziphus, mainly because it takes place at room temperature and low contact time (30 min). This work brings unprecedented results for the use of the species *Ziziphus joazeiro* submitted to adsorptive processes. 

### 3.4. Spectrum FT-IR and Adsorptions Mechanisms 

The bark of *Ziziphus joazeiro* proved to be a good adsorbent, as it showed a good potential in the removal of Pb(II) ions, both in the form treated with 0.5 N NaOH and ethanol (at 80%), and in the natural form, washed with distilled water, showing similar results or even greater than some maximum capacities obtained by other plants of the genus. It was possible to visualize the similarities and differences between the treatments, through the kinetic studies, due to the fact that all the adsorbents reached equilibrium in the last time tested, as well as the equilibrium isotherm of the adsorbent NaOH, reaching equilibrium at the last concentration tested. 

**Figure 4:**
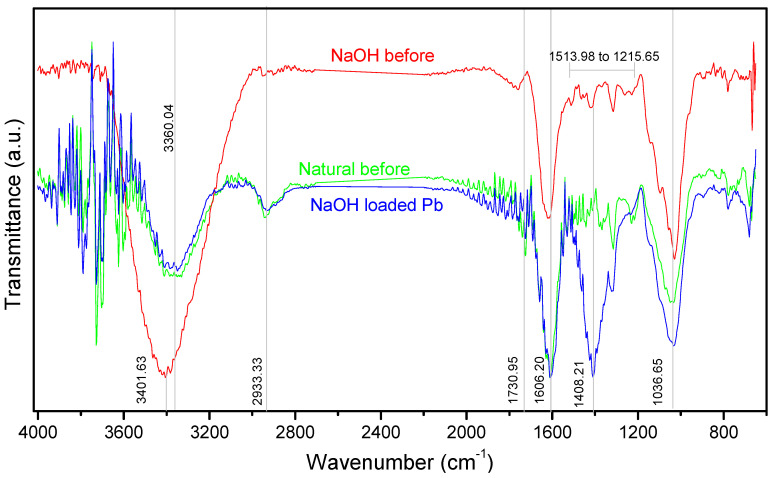
Spectrum FT-IR of Natural and washed with NaOH adsorbents, before and after contact with Pb(II) ions.

To understand the possible interaction sites after the adsorptive process with Pb(II) ions, Figure 4 shows the FT-IR spectrum of the Natural and NaOH samples, before and after contact with metallic ions.

Analyzing the FT-IR spectrum, the peak of 3360.04 cm^−1^ was shifted to 3401.63 cm^−1^, with a significant increase in intensity and width after the alkaline treatment. These peaks are commonly associated with intra- and intermolecular -OH stretching vibration from cellulose, lignin and hemicellulose [81], in addition to saponins [82]; such behavior was also observed in the treatment performed by [12,83]. The 2933.33 cm^−1^ peak referring to the stretching of the C-H bonds of the hydrocarbons became more discreet, while those in the 1200 to 1500 cm^−1^ range showed a number of peaks and a reduction in width. The peak of 1606.20 cm^−1^ can be associated with the structure of the aromatic compounds probably from the lignin structure [50,84], suggesting the permanence of its structure after alkalinization. Similarly, it occurred at 1730.95 cm^−1^ in the C=O stretches related to carboxylic groups, such as carboxylic and ester [12,39,85].

The peak of 1036.65 cm^−1^ refers to the C-O stretches of holocellulose and lignin [81], which may come from ether and phenol [12], or the carboxylic and alcohol groups [86], and can also be attributed to the linkage absorption C-O-C [87] to oligosaccharide present in the structure of saponin [27,88], assuming that this substance has not been completely removed by contact with 0.5 N NaOH, as the alkaline treatment generally induces extensive modifications in the polysaccharides [89,90]. After contact with Pb(II) ions, the enlargement and increase in intensity of this region can be noticed.

The main change after contact with the Pb(II) ions occurred in the appearance of a new intense peak at 1408.21 cm^−1^ that indicates the possibilities of functions such as: C-H vibrations of the aromatic groups [69], the -OH bending vibration of lignin [81], or the stretching of C-H bonds of the alkane and alkyl groups [29]. 

Through visible changes in the spectrum, Pb(II) ions probably interacted with the functional groups -OH, C-O, both of the lignocellulosic structures, mainly lignin that has functional groups that may be involved in complexation reactions with metallic cations, as well as the ion exchange mechanism [91], but apparently they were not affected by the contact with the base due to the low concentration applied (0.5 N); as well as the possibility of interaction with many ether and alcohol groups from the structures of the jujuboside saponin, quite present in the composition of *Z. joazeiro* (between 2 and 10%) [4], its quantification being important to evaluate the effectiveness of treatments applied to the shell. The results of this study are in line with those of plant biomass, in which they associate the hydroxyl, carboxyl and carbonyl groups as potential active sites for interaction with Pb(II) ions [12,37,78,92].

The analysis of the interactions above strengthens the hypothesis of the theoretical and empirical models (Langmuir and Temkin) determined in this study, in which they suggest chemosorption as an adsorptive process, assuming it is by ion exchange or complexation. In addition to the essential characterization of the adsorbent materials by X-ray Fluorescence or scanning electron microscopy (SEM–EDS detector) and Spectroscopy (XRF) to verify and confirm the presence of Pb(II) in the post-contact adsorbent, the determination of ions (calcium, sodium for example) confirms the occurrence of the ion exchange mechanism [93].

Considering chemical adsorption as a reality for this material, we recommend the determination of desorption assays to understand the regenerative capabilities of the adsorbent and metal recovery.

### 3.5. Desorption Studies

Information about the dynamics of desorption in adsorbents is important to the recovery of contaminants and the reusability of the adsorbent, saving on the consumption of adsorbents. Table 6 presents the desorption efficiencies of the NaOH adsorbent samples.

Good desorption efficiencies were verified, with the NaOH adsorbent over 81% within 20 min, and small variations between 10 and 30 min (<3.9%), not justifying the performance of new tests at longer times. The data suggest that the adsorption process is reversible, and hence the adsorbents can be recycled, and the Pb(II) ions recovered for use and incorporation into other production processes. 

## 4. Conclusions

According to experimental data of the adsorption kinetics, all the *Ziziphus joazeiro* bark tested showed the potential to remove Pb(II) ions in aqueous solutions. Among the proposed treatments, the adsorbent modified by 0.5 N NaOH obtained the highest *qexp* (25.5 mg·g^−1^ at 30 min), and the adsorbent washed with ethanol had the least effective performance (20.4 mg·g^−1^ at 60 min), and the maximum removals were 99.9 and 99.6%, respectively. The use of water to wash the bark of *Z. joazeiro* is a good treatment, presenting results similar to the adsorbent washed with NaOH, but with kinetics four times slower. 

The kinetic models pointed to a probable chemisorption for the five samples tested due to the best fit of pseudo-second order and Elovich. Boyd’s model suggests that intraparticle diffusion has its contribution in control and is a limiting step of the adsorption process until the initial minutes of contact for the adsorbents washed with water (16 min), washed by ethanol (8 min) and natural (30 min), due to the orientation of the straight approaching origin.

The Langmuir isotherm fitted better to the experimental data for the NaOH adsorbent, with maximum adsorption capacity equal to 62.5 mg·g^−1^, although the Temkin model partially fitted, both suggesting the occurrence of chemical adsorption, evidenced by the interaction of the Pb(II) ions with the functional groups -OH, C-O in the FT-IR spectrum, with the lignocellulosic structure such as saponin. 

The adsorption process is reversible (>81% at 20 min) and hence the adsorbents can be recycled and the Pb(II) ions recovered for uses in other production processes.

## Figures and Tables

**Table 1:** Chemical and physical modification processes of the adsorbents tested in the research.

Adsorbent	Particle Size (µm)	Proceedings
Natural (N)	1180–300	Raw bark without changes.
Washed with water (WW)		55 g of bark was washed manually with 2 L of distilled water and drying in oven at 103 °C for 22 h.
Washed with ethanol (at 80%) (EE)		10 g of bark was submerged in 100 mL of Ethanol at 80% for 72 h, followed by washing with distilled water and drying in oven at 103 °C for 24 h.
Washed with 0.5 N NaOH (NaOH)		5 g of bark was washed with 125 mL of 0.5 N NaOH solution for 1 h, under 200 rpm stirring, followed by rinsing in 120 mL of distilled water under 200 rpm stirring for 1 h. Drying in oven for 1 h at 50 °C.
Natural powder (NP)	<300	Natural waste less than 300 µm

**Table 2:** Kinetic parameters Pseudo-first order, Pseudo-second order and Elovich model for all adsorbents.

Pseudo-First Order—PFO Model Nonlinear									
Adsorbent	*qexp* (mg·g^−1^)	q_model_ (mg·g^−1^)	Error Relative (%)	R^2^ Adjusted	SSE	χ^2^	RMSE	*k*_1_	
WW	25.0	21.3	14.8	0.851	61.8	8.83	2.62	0.823	
N	21.6	19.0	12.2	0.896	31.6	4.51	1.87	1.433	
EE	20.4	18.7	8.2	0.962	10.7	1.54	1.09	1.412	
NP	22.6	20.8	8.2	0.953	16.2	2.32	1.34	1.886	
NaOH	25.5	24.7	3.3	0.974	11.9	1.98	1.15	0.235	
**Pseudo-Second Order—PSO Model Nonlinear**									
**Adsorbent**	***qexp*** **(mg·g^−1^)**	**q_model_ (mg·g^−1^)**	**Error** **Relative (%)**	**R^2^** **Adjusted**	**SSE**	**χ^2^**	**RMSE**	***k*_2_**	
WW	25.0	22.8	8.9	0.933	27.9	3.99	1.76	0.042	
N	21.6	19.8	8.4	0.942	17.7	2.53	1.40	0.099	
EE	20.4	19.3	5.2	0.986	3.8	0.54	0.65	0.124	
NP	22.6	21.3	6.0	0.971	9.9	1.41	1.04	0.176	
NaOH	25.5	25.9	1.5	0.991	3.9	0.65	0.66	0.017	
**Elovich Model Nonlinear**									
**Adsorbent**	***qexp*** **(mg·g^−1^)**	**q_model_ (mg·g^−1^)**	**Error** **Relative (%)**	**R^2^** **Adjusted**	**SSE**	**χ^2^**	**RMSE**	**β** **(g·mg^−1^)**	**α** **(mg·g^−1^·min^−1^)**
WW	25.0	25,6	2.2	0.994	2.50	0.36	0.53	0.453	9.89 × 10²
N	21.6	21.9	1.4	0.994	1.69	0.24	0.43	0.707	3.09 × 10^4^
EE	20.4	20.8	2.0	0.994	1.69	0.24	0.43	0.939	1.36 × 10^6^
NP	22.6	23.0	1.3	0.998	0.67	0.10	0.27	0.982	2.46 × 10^7^
NaOH	25.5	27.1	6.1	0.968	14.17	2.36	1.25	0.473	3.22 × 10^3^

**Table 3:** Boyd’s Intraparticle Diffusion model parameters for stage I (initial minutes).

Boyd Model					
Adsorbent	B	D (cm^2^·min)	Intercept (b)	R^2^ Adjusted	SSE
WW	0.058	1.30 × 10^−4^	0.3218	0.999	0.0003
N	0.042	9.40 × 10^−5^	0.6335	0.985	0.0113
EE	0.111	2.46 × 10^−4^	0.6454	0.990	0.0015
NP	0.039	1.41 × 10^−5^	1.0270	0.947	0.0069
NaOH	0.13	2.52 × 10^−4^	0.0782	0.907	0.0471

**Table 4:** Parameters of equilibrium isotherm models applied to the adsorbent NaOH.

Model	Parameters		R^2^ Adjusted	SSE	χ^2^	RMSE	Relative Error (%)
	*qmax.* Experimental (mg·g^−1^)	58.3					
Langmuir	*qmax* (mg·g^−1^)	62.5	0.983	90.9	7.6	2.55	7.3
	K_L_ (L·mg^−1^)	0.04					
Freundlich	K_F_ (mg·g^−1^)(L·mg^−1^)^1/n^	10.86	0.871	705.6	58.8	7.10	-
	1/n	0.30					
	n	3.34					
Temkin	K_T_ (L·mg^−1^)	0.66	0.959	223.1	18.6	-	-
	b (J/mol)	220.80					

**Table 5:** Maximum adsorption capacity of Pb(II) ions from different adsorbents from Ziziphus genus by Langmuir model.

Types of Adsorbents	Modifications	Experimental Conditions			*qmax.* Langmuir (mg·g^−1^)	References
		pH	°C	Time (min)		
Jujuba seeds	Row	5	30	60	6.65	[30]
	H_2_SO_4_ and Ultrasound			90	119.8	
*Z. spina-christi*	Citric acid	6	-	60	9.06	[12]
Jujube pit powder	Pyrolisis	6	25	30	137.1	[29]
*Ziziphus jojoba* leaves	HNO_3_ CaCl_2_	6	50	120	58.47	[21]
*Jujuba stone*	H_2_SO_4_	6	25	10	60.44	[78]
*Z. spina christi leaves* ash	600°	5	20	30	7.23	[79]
*Z. mauritiana* seeds stones	-	4	-	180	2.47	[80]
*Ziziphus joazeiro* bark	0.5 N NaOH	5.5	24–25	30	58.3	Present study

**Table 6:** Desorption efficiency of Pb(II) ions from NaOH adsorbent with 0.1 M HCl.

Adsorbents	Desorption Efficiency (%)		
Washed by NaOH	10 min	20 min	30 min
	78.1	81.2	81.3

## Data Availability

Not applicable.

## Appendix A

**Table A1:** Error function equations used to evaluate the best fit of kinetic and isothermal models.

Function	Equations	Parameters
R^2^ Adjusted	${R^{2}}_{\mathrm{adj}}=1-\frac{\left(1-R^{2}\right)\left(N-1\right)}{N-P-1}$	P: Number of parameter models N: Size sample (number data experiment) R^2^: Determination coefficient *qexp*: Adsorption capacity experiment (mg·g^−1^) q_model_: Adsorption capacity calculated by model
Relative error (%)	$\mathrm{RE}\%=\frac{qexp-q_{\mathrm{model}}}{qexp}\times 100$	
Sum of square error	$\mathrm{SSE}=\overset{N}{\underset{i=1}{\sum }}{\left(qexp-q_{\mathrm{model}}\right)}^{2}$	
Mean squared errors	$\mathrm{MSE}=\frac{\mathrm{SSE}}{N}$	
Root mean squared errors	$\mathrm{RMSE}=\sqrt{\mathrm{MSE}}$	
Chi-square	$\chi^{2}=\overset{N}{\underset{i=1}{\sum }}\frac{{\left(qexp-q_{\mathrm{model}}\right)}^{2}}{qexp}$	
